# *Candida* *tropicalis*: An Emerging Opportunistic Pathogen at the Interface of Virulence, Antifungal Resistance, and Host Immune Interactions

**DOI:** 10.3390/microorganisms14092038

**Published:** 2026-09-12

**Authors:** Manuela Gómez-Gaviria, Dario A. Baruch-Martínez, Héctor M. Mora-Montes

**Affiliations:** Departamento de Biología, División de Ciencias Naturales y Exactas, Campus Guanajuato, Universidad de Guanajuato, Noria Alta s/n, Col. Noria Alta, Guanajuato C.P. 36050, Mexico; mgomezg@ugto.mx (M.G.-G.); da.baruchmartinez@ugto.mx (D.A.B.-M.)

**Keywords:** biofilms, cell wall, immune evasion, morphogenesis, non-*albicans Candida*, virulence factors

## Abstract

*Candida tropicalis* has emerged as one of the most clinically relevant non-*albicans Candida* species, owing to its increasing global prevalence, high mortality associated with invasive infections, and rising rates of antifungal resistance. Although traditionally considered an opportunistic pathogen, growing evidence indicates that its remarkable adaptive capacity is driven by the coordinated regulation of multiple biological processes that promote host colonization, persistence, and immune evasion. In this review, we summarize current knowledge on the epidemiology, biology, genomic organization, virulence factors, and host–pathogen interactions of *C. tropicalis*. Particular emphasis is placed on recent advances in comparative genomics and functional studies that have expanded our understanding of the molecular determinants underlying adhesion, biofilm formation, morphogenesis, extracellular hydrolytic enzyme production, thermotolerance, cell wall remodeling, and immune evasion. We also integrate orthology analyses identifying putative *C. tropicalis* homologs of well-characterized *Candida albicans* virulence genes, highlighting the evolutionary conservation of key pathogenic mechanisms while emphasizing species-specific adaptations that remain functionally unexplored. Finally, we discuss current knowledge of antifungal resistance and the emerging relationship between genomic plasticity, stress adaptation, and pathogenicity. Together, this review provides an updated and comprehensive overview of the biological mechanisms that contribute to the success of *C. tropicalis* as an emerging opportunistic pathogen and identifies key areas requiring further investigation to improve diagnosis, treatment, and the development of novel antifungal strategies.

## 1. Introduction

Invasive fungal infections represent a growing global public health problem, particularly among immunocompromised populations and in hospital settings [1]. It is estimated that these infections are responsible for more than 1.5 million deaths annually, with a disproportionate impact on regions where marginalization and limited access to health services are prevalent [2,3]. However, this figure, while alarming, only partially reflects the true magnitude of the problem, as mortality alone does not capture the complexity of the fungal burden in the various affected populations [1,4].

In this context, the genus *Candida* stands as one of the primary causative agents of fungal infections in humans, encompassing a broad clinical spectrum ranging from superficial mucocutaneous infections to life-threatening invasive candidiasis [5,6]. Historically, *Candida albicans* has been the predominant species in the clinical setting; however, in recent decades, a significant shift in the epidemiology of these infections has been observed, characterized by a sustained increase in the prevalence of non-*albicans* species [7,8]. This shift has been associated with various factors, including the widespread use of antifungals, selective pressure in hospital settings, and the growth of vulnerable populations [7].

In this epidemiological context, *Candida tropicalis* has emerged as one of the most clinically significant non-*albicans* species, particularly in tropical and subtropical regions, although its distribution is now global [9,10]. This species is recognized as a major cause of candidemia and invasive infections, with a notable association with patients with hematologic malignancies, neutropenia, and other immunosuppressive conditions [9]. Its clinical relevance stems not only from its increasing prevalence but also from its high capacity for systemic dissemination and the high associated mortality rates, which can reach up to 55–60% in invasive infections [10]. Furthermore, in recent years, a concerning increase in antifungal resistance has been documented, particularly to azole compounds, which has contributed to its positioning as a high-impact emerging pathogen [11]. This epidemiological shift, together with its distinctive biological characteristics, underscores the need for a deeper understanding of the mechanisms regulating its virulence, adaptation to the host, and response to antifungal pressure [12].

The objective of this review is to provide an updated and integrative analysis of the biological and clinical features of *C. tropicalis*, with particular emphasis on the molecular determinants underlying its pathogenicity and adaptation to the host. In addition to critically examining recent advances in epidemiology, virulence, host–pathogen interactions, antifungal resistance, and diagnosis, we integrate a comparative BLASTp-based orthology analysis of major virulence-associated proteins characterized in *C. albicans* to explore the conservation of these determinants in *C. tropicalis*. By distinguishing mechanisms experimentally characterized in *C. tropicalis* from those primarily inferred through comparative genomic evidence, this review highlights species-specific features, identifies current knowledge gaps, and provides an updated framework for understanding the emergence and pathogenic success of this clinically important species.

## 2. Biological Aspects

### 2.1. General Aspects

**Figure 1:**
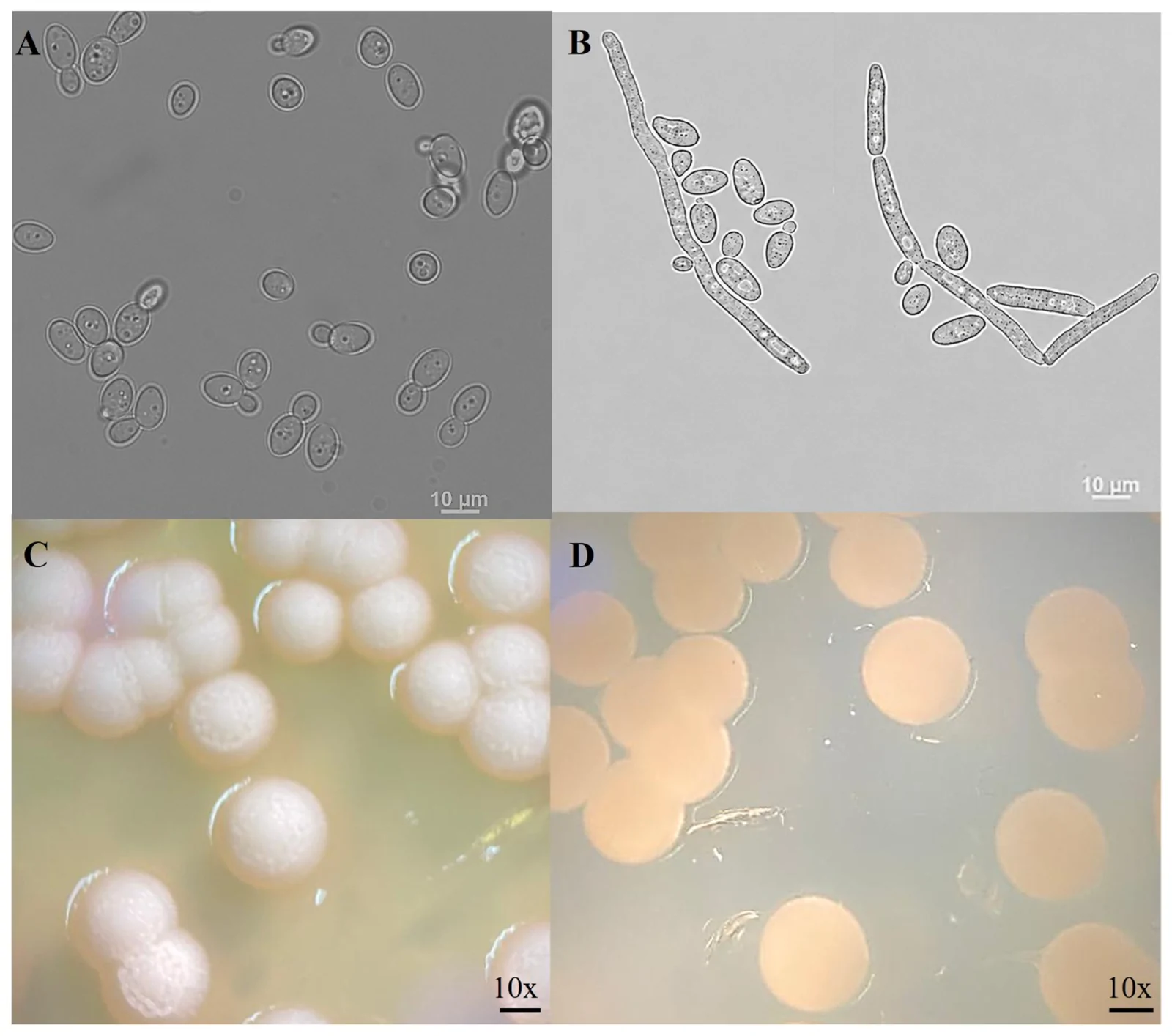
Morphological characteristics of *Candida tropicalis* ATCC MYA-3404. (**A**) Yeast cells grown in Sabouraud dextrose medium at 28 °C and observed by light microscopy using a 60× objective, showing the characteristic oval-to-ovoid budding morphology. (**B**) Representative filamentous morphologies induced at 37 °C in yeast nitrogen base (YNB) medium supplemented with fetal bovine serum and a low glucose concentration, showing both true hyphae and pseudohyphae. True hyphae are characterized by elongated tubular structures with parallel-sided walls and no evident constrictions at the septal junctions, whereas pseudohyphae consist of chains of elongated cells displaying constrictions between adjacent cells. Scale bars = 10 µm. (**C**,**D**) Representative colonies of *C. tropicalis* ATCC MYA-3404 grown on Sabouraud dextrose agar, exhibiting the characteristic white-to-cream coloration and creamy texture, with slightly wrinkled (**C**) and smooth (**D**) colony surfaces. Images in panels (**C**,**D**) were acquired at 10× magnification.

*C. tropicalis* is an ascomycetous yeast belonging to the order Saccharomycetales and represents one of the species most closely related phylogenetically to *C. albicans* [13]. It is a diploid species, with its nuclear genome organized into homologous chromosome pairs, a feature that has important implications for its genetic diversity and genomic plasticity. Since its initial description as *Oidium tropicale* in the early twentieth century, this species has gained increasing medical importance owing to its widespread geographic distribution and its association with human infections of varying severity [14,15]. Similar to *C. albicans* and *Candida dubliniensis*, *C. tropicalis* produces yeast cells, pseudohyphae, and true hyphae, a characteristic closely associated with its phenotypic plasticity and its ability to adapt to diverse environmental and host-associated conditions (Figure 1) [16,17]. In addition, this species exhibits phenotypic switching and a remarkable ability to respond to a wide range of environmental cues, features that contribute to its success as an opportunistic microorganism [12].

*C. tropicalis* is part of the normal human microbiota and can be isolated from the skin and the mucosal surfaces of the gastrointestinal, genitourinary, and respiratory tracts [18,19,20]. Under physiological conditions, it establishes a commensal relationship with the host; however, alterations in the microbiota, disruption of epithelial barriers, or immunosuppressive states can promote the transition from a commensal organism to an invasive pathogen [12,21]. This ability to alternate between these two lifestyles is considered one of the key features underlying its success as an opportunistic pathogen [22]. Furthermore, *C. tropicalis* exhibits several biological characteristics that distinguish it from other members of the genus, including pronounced osmotolerance, the ability to grow under high NaCl concentrations, and remarkable metabolic versatility. These traits promote its survival in both environmental niches and host-associated habitats and have also attracted interest because of their potential biotechnological applications [22,23,24].

### 2.2. Morphology

Similar to other pathogenic members of the genus, *C. tropicalis* exhibits remarkable morphological plasticity and is capable of growing in different cellular forms, including yeast cells, pseudohyphae, and true hyphae [12,25]. Yeast cells are spherical to ovoid and reproduce by budding (Figure 1), whereas pseudohyphae consist of elongated cells with constrictions at the septal junctions, representing an intermediate morphology between yeast cells and true hyphae. In contrast, true hyphae are characterized by parallel-sided walls and the absence of constrictions at the septal junctions, features that distinguish them from pseudohyphal structures [22,25]. In addition, when grown on Sabouraud dextrose agar, *C. tropicalis* forms circular, white-to-cream colonies with a creamy texture and a smooth to slightly wrinkled surface (Figure 1), which are characteristic macroscopic features of this species [12,15,26].

The transition between these morphological forms is regulated by multiple environmental and host-derived signals, including changes in temperature, pH, nutrient availability, carbon dioxide concentration, and the presence of specific molecules such as N-acetylglucosamine [12,27,28]. These environmental cues can shift the balance between yeast and filamentous growth. Host-associated conditions, including temperatures near 37 °C, elevated CO_2_ levels, and the presence of serum or N-acetylglucosamine, generally favor the development of pseudohyphae and hyphae, whereas nutrient composition and availability can either promote or restrict filamentation depending on the growth conditions [12,27,29]. Extracellular pH also influences morphogenesis, with changes in pH being sensed by regulatory pathways that modulate the expression of genes involved in polarized growth and cell-wall remodeling. Importantly, these signals do not act independently but are integrated through interconnected signaling networks, allowing *C. tropicalis* to adjust its morphology according to the surrounding microenvironment [12]. This ability to respond to diverse stimuli confers considerable physiological versatility, enabling *C. tropicalis* to colonize different niches and adapt to changing environmental conditions.

In addition to the transition between yeast and filamentous forms, *C. tropicalis* undergoes phenotypic switching, generating cell populations with distinct morphological and physiological characteristics. Initially, white and opaque phenotypes, analogous to those described in *C. albicans*, were identified. However, the subsequent discovery of a third phenotypic state, termed gray, revealed the existence of a tristable phenotypic switching system [27,30]. These three phenotypic states differ markedly in colony morphology, cellular architecture, metabolic activity, mating competence, and virulence-associated traits. White cells are generally round to oval budding yeast cells that exhibit high proliferative capacity and represent the predominant morphology under standard laboratory conditions. Opaque cells are larger and more elongated, display altered cell wall properties, and exhibit enhanced mating competency, reflecting extensive transcriptional reprogramming. Gray cells exhibit intermediate morphological characteristics but possess a unique transcriptional profile that is distinct from both white and opaque cells, indicating that they represent an independent developmental state rather than a simple transitional intermediate [29,30].

Phenotypic switching is primarily controlled by the master transcriptional regulator Wor1, together with an interconnected regulatory network involving additional transcription factors that integrate environmental and physiological signals. Changes in temperature, nutrient availability, carbon source, oxygen tension, and host-associated stimuli can influence switching frequencies, allowing fungal populations to rapidly adapt to fluctuating environmental conditions [30,31]. Importantly, each phenotypic state exhibits distinct patterns of gene expression, suggesting extensive metabolic specialization and niche-specific adaptation.

Beyond its role in cellular differentiation, phenotypic switching has important implications for pathogenicity. Individual phenotypic states differ in their ability to colonize host tissues, form biofilms, undergo filamentation, interact with immune cells, and respond to environmental stresses. Consequently, maintenance of multiple stable phenotypes within a single population increases the adaptive potential of *C. tropicalis*, enabling rapid responses to diverse host environments and contributing to its persistence during infection [12,29,32,33].

### 2.3. Cell Wall Composition and Organization

The *C. tropicalis* cell wall is a dynamic and highly organized structure that plays essential roles in maintaining cellular integrity, protecting against adverse environmental conditions, and mediating interactions with the host. Similar to other pathogenic species of the *Candida* genus, the cell wall is organized into two major layers: an inner structural layer and an outer fibrillar layer [34]. The inner layer is primarily composed of chitin and β-glucans, particularly β-1,3-glucan and β-1,6-glucan, which provide mechanical strength and structural support to the fungal cell. In contrast, the outer layer is enriched in highly glycosylated mannoproteins that are covalently linked to the glucan matrix and constitute the fungal surface directly exposed to the extracellular environment [34,35].

Although the overall architecture of the cell wall is conserved among pathogenic *Candida* species, several studies have demonstrated that *C. tropicalis* possesses distinctive structural features. Biochemical analyses have shown that glucans account for approximately 60% of the cell wall dry weight, whereas mannans represent nearly 36% and chitin only 2–3% [35]. The *C. tropicalis* mannans form complex structures composed of mannose residues linked through α-1,2-, α-1,6-, and β-1,2-glycosidic bonds, the latter being recognized as important antigenic determinants in pathogenic *Candida* species [36]. In addition, *N*-linked mannans may be modified with phosphomannan, a structure consisting of β-1,2-linked oligomannosides attached through phosphodiester bonds. Interestingly, *C. tropicalis* has been reported to contain higher phosphomannan levels than *C. albicans*, a feature that may influence the physicochemical properties of the fungal surface as well as its interactions with the host immune system [37]. Similar to *C. albicans*, most β-1,3-glucan and chitin are localized within the inner layers of the *C. tropicalis* cell wall and remain partially masked by the outer mannan-rich layer [34]. This spatial organization not only contributes to cell wall stability but also plays a critical role in modulating immune recognition by the host [10,34].

Beyond its structural role, the cell wall represents the primary interface between *C. tropicalis* and the host. Dynamic remodeling of its polysaccharide architecture continuously modifies the accessibility of pathogen-associated molecular patterns (PAMPs), thereby regulating fungal recognition by pattern recognition receptors such as Dectin-1, Toll-like receptors, the mannose receptor, and complement receptor 3 (CR3). Consequently, alterations in cell wall composition directly influence cytokine production, phagocytosis, complement activation, and the magnitude of the inflammatory response, making cell wall remodeling a central mechanism of immune adaptation and virulence [10,34,35].

In addition to structural polysaccharides, the *C. tropicalis* cell wall contains numerous cell surface-associated proteins, many of which are attached to the wall through glycosylphosphatidylinositol anchors [38]. These proteins participate in fundamental processes such as adhesion to host tissues and abiotic surfaces, nutrient acquisition, cell wall maintenance, and adaptation to environmental stress.

These proteins are extensively modified by both *N*-linked and *O*-linked glycosylation, post-translational modifications that contribute to the organization and biological properties of the *C. tropicalis* cell wall. Importantly, functional studies in *C. tropicalis* have demonstrated that disruption of specific components of the glycosylation machinery produces distinct alterations in cell wall architecture and host–fungus interactions [34,35,39]. The *OCH1*disruption, a gene that encodes an α-1,6-mannosyltransferase required for the elaboration of the outer chain of *N*-linked mannans, results in major changes in cell wall organization and alters the exposure of immunologically relevant wall components [34]. Likewise, disruption of genes involved in protein *O*-mannosylation, particularly members of the *PMT* family, affects cell morphology, cell wall integrity, filamentation, biofilm formation, and interaction with host immune cells [34,35]. These findings demonstrate that protein mannosylation in *C. tropicalis* is not merely a conserved structural process but directly contributes to several phenotypes associated with pathogenicity [34].

A particularly distinctive feature of the *C. tropicalis* cell surface is its phosphomannan content, which has been reported to be higher than that of *C. albicans* [37]. Phosphomannan contributes to the surface charge and physicochemical properties of the fungal cell and can influence its interaction with host immune receptors. Alterations in mannan structure consequently modify the accessibility of inner-wall β-glucans and chitin and change the pattern of recognition by innate immune receptors. Indeed, studies using *C. tropicalis* mutants defective in *N*- and *O*-linked mannosylation have demonstrated altered recognition by human macrophages and differential production of TNF-α, IL-6, and IL-10, providing direct evidence that cell wall glycosylation modulates the immunological properties of this species [34,35].

The composition and organization of the *C. tropicalis* cell wall are also dynamically remodeled in response to environmental and antifungal stress. Recent studies have shown that this remodeling can differ according to the antifungal susceptibility phenotype. Fluconazole-resistant *C. tropicalis* isolates exhibit altered cell wall organization, including reduced surface exposure of β-1,3-glucan, together with transcriptional changes in genes associated with cell wall biosynthesis and remodeling [34,35,40]. This phenotype may simultaneously contribute to adaptation to antifungal pressure and reduced recognition by β-glucan-sensing immune receptors such as Dectin-1 [40]. Thus, rather than representing only a conserved stress response, cell wall remodeling in *C. tropicalis* is closely interconnected with antifungal adaptation, immune recognition, and pathogenicity [33,34].

This remodeling process is coordinated by conserved cell wall integrity (CWI) signaling pathways that continuously monitor cell wall architecture and activate compensatory biosynthetic mechanisms in response to stress [41]. Through this regulatory network, *C. tropicalis* rapidly adjusts the relative abundance of glucans, chitin, mannans, and cell wall proteins, allowing the fungus to preserve structural integrity while adapting to changes in pH, temperature, nutrient availability, oxidative stress, antifungal drugs, and host immune pressure. Therefore, the fungal cell wall should be regarded not as a static protective barrier but as a highly dynamic organelle that integrates environmental sensing, stress adaptation, and virulence [33,41].

### 2.4. Genome

**Figure 2:**
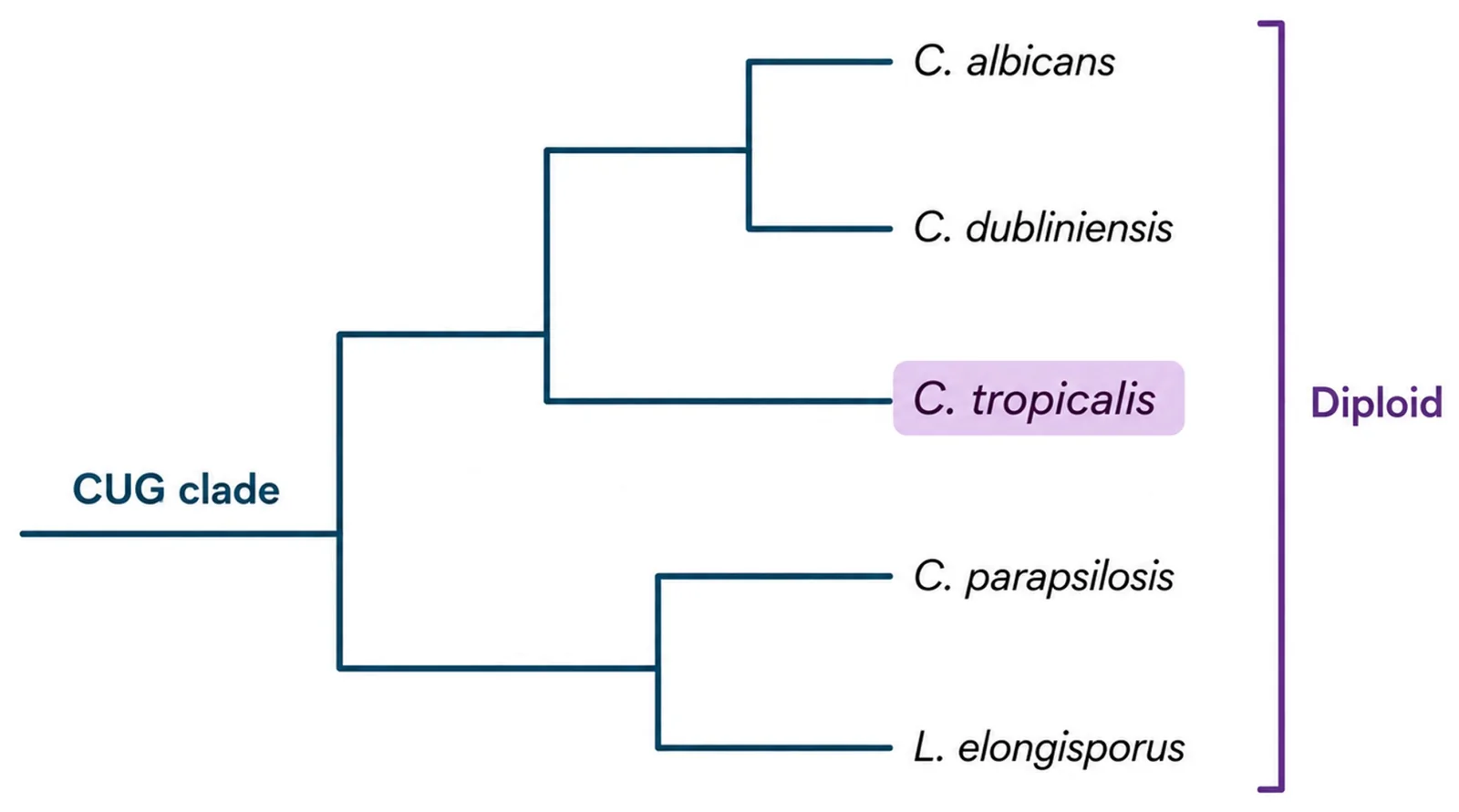
Schematic representation of the phylogenetic relationships of *Candida tropicalis* within the diploid members of the CUG clade. The diagram illustrates the phylogenetic position of *Candida tropicalis* relative to *Candida albicans*, *Candida dubliniensis*, *Candida parapsilosis*, and *Lodderomyces elongisporus*. *C. tropicalis* is highlighted to emphasize its position within this group. The topology represents a simplified depiction based on previously reported phylogenetic relationships among species of the CUG clade.

*C. tropicalis* is a diploid yeast belonging to the CTG (or CUG-Ser1) clade, a group of ascomycetes characterized by the alternative translation of the CUG codon as serine rather than leucine (Figure 2). This distinctive genetic feature is shared with other pathogenic *Candida* species, including *C. albicans* and *C. parapsilosis*. The first reference genome assembly of *C. tropicalis* (strain MYA-3404) was published in 2009, revealing a genome of approximately 14.5 Mb, with a GC content of about 33% and approximately 6250 protein-coding genes (https://www.ncbi.nlm.nih.gov/datasets/taxonomy/294747/, accessed on 2 May 2026) [13]. Subsequent genomic studies and newly available genome assemblies deposited in public databases have confirmed that the genome size of *C. tropicalis* ranges from 14.5 to 15.5 Mb, depending on the strain analyzed [12,13].

The *C. tropicalis* nuclear genome is organized into seven pairs of chromosomes and exhibits a high degree of heterozygosity, a feature that contributes substantially to its adaptive potential [40,42]. Whole-genome sequencing analyses have shown that most isolates harbor between two and six heterozygous variants per kilobase, although some strains display considerably higher levels of genetic variation [12,43]. This diversity represents an important source of genomic plasticity and promotes adaptation to diverse ecological niches and environmental stresses. In addition, frequent loss of heterozygosity (LOH) events have been described. These events generate homozygous regions from initially heterozygous genomes and may contribute to evolutionary adaptation, the emergence of resistant phenotypes, and the optimization of cellular fitness under selective pressures [28,44].

Although *C. tropicalis* was long considered a predominantly asexual species, more recent studies have demonstrated the existence of a functional mating system. Fusion between diploid cells of opposite mating types (a and α) can generate tetraploid a/α cells, a process regulated by the white-to-opaque phenotypic switching program, as only opaque cells are mating competent [27,30,45]. Unlike many other eukaryotes, meiosis has not been described in *C. tropicalis*. Instead, tetraploid cells can undergo a parasexual cycle characterized by progressive ploidy reduction through chromosome loss, eventually returning to the diploid state after approximately 240 generations [28,46]. This process generates novel genetic combinations through chromosomal recombination events and represents an important source of genetic diversity [10,47].

Genomic plasticity derived from this parasexual cycle promotes the emergence of aneuploidies, polyploidies, and additional LOH events capable of altering gene expression patterns and protein production [28,45]. These genomic alterations are considered adaptive mechanisms that enable cells to respond to a variety of stress conditions. Indeed, environmental stresses such as heat shock, ultraviolet radiation, and growth on alternative carbon sources, including L-sorbose and D-arabinose, have been shown to induce changes in ploidy and promote chromosomal rearrangements [48,49,50]. Consequently, karyotypic variation may arise even during host colonization or infection, providing a selective advantage under changing environmental conditions [12].

Population genomic studies have revealed a complex genetic structure within the species [43,44]. Comparative analyses of clinical and environmental isolates collected from different geographic regions have identified multiple globally distributed genetic clades, as well as hybrid lineages resulting from recombination events between divergent populations [43,51]. Interestingly, environmental and clinical isolates do not form completely distinct groups, suggesting the existence of environmental reservoirs capable of contributing to the emergence and dissemination of potentially pathogenic strains. These findings highlight the importance of integrating ecological and genomic approaches to better understand the evolution and epidemiology of *C. tropicalis* [44].

High-throughput sequencing approaches have also enabled the identification of genes and regulatory networks involved in fundamental biological processes, including cellular adhesion, biofilm formation, morphological transitions, stress responses, and host interactions [12,44]. Comparative genomic analyses have further revealed the presence of numerous gene families encoding cell surface proteins, hydrolytic enzymes, and factors associated with cell wall integrity, many of which are homologous to virulence genes previously characterized in *C. albicans* [13]. These observations underscore the complexity of the molecular strategies employed by *C. tropicalis* during colonization and infection [12,44].

More recently, whole-genome sequencing and transcriptomic studies have identified specific lineages associated with azole resistance and enhanced adaptive potential [10,44]. These isolates harbor recurrent mutations in genes involved in ergosterol biosynthesis, particularly *ERG11*, as well as alterations in genes associated with adhesion, biofilm development, and stress adaptation [9,44]. Furthermore, distinct transcriptional profiles have been observed, suggesting a close relationship between genome evolution, host adaptation, and the development of antifungal resistance [44]. Collectively, these findings indicate that genomic plasticity is one of the major determinants of the adaptive capacity and pathogenic potential of *C. tropicalis* [28,43,44].

## 3. Virulence Factors

The ability of *C. tropicalis* to establish infection relies on the coordinated action of multiple virulence determinants that enable the fungus to adhere to host tissues, invade diverse anatomical niches, adapt to changing environmental conditions, evade immune defenses, and persist throughout the course of infection [52,53]. Unlike obligate pathogens, *C. tropicalis* is an opportunistic microorganism whose pathogenic potential is not governed by a single virulence trait but rather by the dynamic interplay of multiple biological processes that are tightly regulated in response to both host- and environment-derived signals. This remarkable adaptive capacity largely explains its emergence as one of the leading non-*albicans Candida* species responsible for invasive candidiasis and candidemia worldwide [33].

Over the past decade, advances in comparative genomics, transcriptomics, and functional genomics have substantially expanded our understanding of the molecular basis underlying *C. tropicalis* virulence [12,33]. These approaches have identified numerous genes and regulatory networks involved in essential pathogenic processes, including host cell adhesion, biofilm development, morphological transitions between yeast and filamentous forms, secretion of extracellular hydrolytic enzymes, nutrient acquisition, stress adaptation, thermotolerance, and immune evasion [12,54]. Many of these determinants are homologous to virulence factors previously characterized in *Candida albicans*, whereas others appear to represent species-specific adaptations that may contribute to the distinctive epidemiological and clinical features of *C. tropicalis* infections [12,32,55,56].

Although several virulence factors have been experimentally characterized in *C. tropicalis*, a substantial proportion of genes potentially involved in pathogenicity have been inferred through comparative genomic analyses based on their high sequence similarity to well-characterized virulence determinants in *C. albicans*. To complement the available experimental evidence, we performed an orthology analysis using BLASTp (version + 2.17.0) with established *C. albicans* virulence proteins as reference sequences. This analysis identified putative orthologs in *C. tropicalis* displaying high sequence similarity to genes associated with adhesion, biofilm formation, morphological switching, immune evasion, extracellular hydrolytic enzyme production, and stress adaptation (Table 1). These findings indicate that a substantial proportion of the molecular repertoire underlying virulence is evolutionarily conserved between the two species, although the specific contribution of many of these genes to *C. tropicalis* pathogenicity remains to be experimentally validated.

The following sections summarize the major virulence factors described in *C. tropicalis*, integrating current experimental evidence with insights derived from comparative genomic analyses. This integrative approach provides a broader understanding of the molecular mechanisms governing host colonization, tissue invasion, immune modulation, persistence, and environmental adaptation, thereby offering a comprehensive overview of the pathogenic strategies employed by this clinically important fungal pathogen.

### 3.1. Cellular Adhesion

Cellular adhesion represents the initial step in the establishment of *C. tropicalis* infection and is considered one of the major determinants of its pathogenicity [16,32]. This process enables the fungus to colonize mucosal surfaces, persist within diverse anatomical niches, and initiate biofilm development, thereby facilitating subsequent tissue invasion and systemic dissemination [12,57]. Host interaction is primarily mediated by cell wall-associated surface proteins known as adhesins, which recognize receptors expressed on epithelial and endothelial cells, extracellular matrix components, and abiotic materials used in medical devices [56,58,59]. Collectively, these interactions allow *C. tropicalis* to establish stable attachment to host tissues while dynamically adapting to microenvironmental changes encountered during infection [54].

Several studies have demonstrated that *C. tropicalis* is among the most adherent non-*albicans Candida* species to host cells [12,16]. In vitro assays have shown that this species exhibits moderate adherence to human buccal epithelial cells, being surpassed only by *C. albicans* [60,61]. Similarly, its adherence to HeLa cells is comparable to that observed for *C. albicans* [62]. However, the adhesive capacity of *C. tropicalis* is highly dependent on the host cell type. For example, *C. tropicalis* displays significantly greater adherence to intestinal Caco-2 cells than to cervical HeLa cells or urinary tract-derived TCC-SUP cells [63]. Likewise, clinical isolates have been reported to adhere more efficiently to HeLa cells than to kidney-derived Vero cells [64]. Interestingly, distinct adhesion patterns have also been observed, with yeast cells forming compact aggregates on HeLa monolayers, whereas a more diffuse distribution predominates on Vero cells. These findings indicate that the interaction between *C. tropicalis* and host cells is strongly cell type-dependent and likely reflects differences in receptor availability, as well as variation in the expression of fungal adhesins and other cell surface components [32,64].

At the molecular level, adhesion in *C. tropicalis* is primarily mediated by highly glycosylated cell wall proteins that are covalently anchored through glycosylphosphatidylinositol (GPI) moieties. Although the adhesin repertoire has been extensively characterized in *C. albicans*, knowledge of these proteins in *C. tropicalis* remains comparatively limited [34,38,65,66]. Nevertheless, comparative genomic studies indicate a high degree of conservation between both species for numerous adhesion-associated determinants. Consistent with these observations, our BLASTp orthology analysis identified putative orthologs of several well-characterized *C. albicans* adhesins, including proteins homologous to Als1/Als3, Als5, Eap1/Hwp1, Mp65, Phr1, Ecm33, Int1, and Iff4 (Table 1).

Among the conserved adhesins, the Agglutinin-Like Sequence (Als) protein family represents the best-characterized group of adhesion proteins in pathogenic *Candida* species [67,68]. These cell wall glycoproteins display a highly conserved modular architecture consisting of an N-terminal signal peptide, an immunoglobulin-like ligand-binding domain, a central region rich in tandem repeats, and a C-terminal domain containing the GPI anchoring signal that covalently links the protein to the fungal cell wall [68]. This structural organization promotes optimal surface exposure of the adhesive domains, enabling interactions with a broad spectrum of host ligands, including epithelial and endothelial cells as well as extracellular matrix proteins. Beyond mediating adhesion, Als proteins also contribute to cellular aggregation, biofilm development, and colonization of multiple anatomical niches, highlighting their central role in *Candida* pathogenicity [67,69,70].

Although the *ALS* gene family has been extensively characterized in *C. albicans*, recent studies have demonstrated that *C. tropicalis* possesses a more complex adhesin repertoire than previously recognized [66]. Comparative genomic analyses have identified at least 13 *ALS* loci distributed throughout the *C. tropicalis* genome, exceeding the eight *ALS* genes described in *C. albicans* and suggesting that this gene family has undergone expansion during the evolution of *C. tropicalis* [66]. Furthermore, these genes exhibit substantial variation in both gene length and the number of tandem repeat sequences, structural features that may influence the ligand-binding properties of Als proteins and contribute to the differences in adhesive capacity observed among clinical and environmental isolates [66].

Consistent with these findings, our BLASTp orthology analysis identified *C. tropicalis* proteins displaying high sequence similarity to the *C. albicans* adhesins Als1/Als3 and Als5, further supporting the evolutionary conservation of adhesion mechanisms between both species (Table 1). However, the expansion of the *ALS* family in *C. tropicalis* suggests that its adhesin repertoire likely extends beyond that inferred solely from comparisons with *C. albicans*, highlighting the need for functional characterization of these genes. Furthermore, expression of individual *ALS* gene family members is differentially regulated in response to environmental conditions and the physiological state of the fungus, indicating that distinct adhesins may contribute to specific stages of host colonization and infection [66].

In addition to Als proteins, several other putative adhesins identified in *C. tropicalis* are likely to play important roles during infection. Mp65, Phr1, and Ecm33 are cell wall proteins characterized in *C. albicans*. In this species, Mp65 has been associated with cellular adhesion, cell wall organization, and immunogenicity; Phr1 is a pH-regulated remodeling enzyme involved in morphogenesis and cell wall integrity; and Ecm33 contributes to cell wall organization and the proper surface localization of cell wall proteins [12,71,72]. In *C. tropicalis*, orthologs of these proteins have been identified, and proteomic studies have detected Mp65 and Phr1 among cell-associated or secreted proteins, while *PHR1* expression has also been associated with morphological transitions. However, their specific contributions to adhesion and virulence in *C. tropicalis* remain less extensively characterized than in *C. albicans* [73,74,75].

Hwp1 and Hyr1 are other cell wall proteins related to *C. albicans* virulence. These have been described in *C. tropicalis*, although the available functional evidence is more limited. *HWP1* expression has been reported in *C. tropicalis*, suggesting a potential contribution to adhesion and biofilm-associated processes [76,77]. Nevertheless, the *C. tropicalis* protein shows considerable divergence from the extensively characterized *C. albicans* Hwp1, and its precise function remains to be established [76]. Members of the *IFF/HYR* gene family are also present in *C. tropicalis*, and Hyr1-related proteins have been associated with interactions with host components, including extracellular matrix proteins and high-molecular-weight kininogen, supporting a potential role in host–fungus interactions [12]. Likewise, Int1 and Iff4 have been implicated in adhesion, filamentation, and virulence primarily in other *Candida* species, and their specific roles in *C. tropicalis* require further functional characterization [12,78].

The expression of many of these adhesins is not constitutive but is dynamically regulated in response to environmental and host-derived signals. Factors such as nutrient availability, pH, osmolarity, temperature, surface contact, morphological switching, and exposure to oxidative stress or antifungal agents can modulate both gene expression and the surface exposure of adhesins, thereby regulating the adhesive capacity of the fungus [32,66]. In *C. albicans*, transcriptional regulators including Bcr1, Efg1, Tec1, and Brg1 coordinate the expression of numerous genes involved in adhesion and biofilm development [12,79,80]. Consistent with these observations, our orthology analysis identified homologs of several of these regulatory proteins in *C. tropicalis* (Table 1), suggesting that conserved regulatory networks may govern these processes, although their precise functions in this species remain to be experimentally established.

Beyond its ability to adhere to host cells, *C. tropicalis* exhibits a remarkable capacity to colonize abiotic surfaces, representing the initial step in biofilm formation on medical devices [16,32]. Adhesion to biomaterials is influenced by both the physicochemical properties of the substrate and the characteristics of the fungal cell wall. Surface roughness, porosity, and hydrophobicity favor fungal colonization, whereas adhesins and the hydrophobic properties of the fungal cell surface promote stable attachment to these materials [81]. Clinically, this characteristic is highly relevant because of the strong association between *C. tropicalis* and device-related infections. This species accounts for approximately 3.8% of catheter-related bloodstream infections in pediatric patients and 1.3% in adults [82]. Moreover, catheter-derived isolates display greater adherence to polystyrene microspheres than other *Candida* species [83]. In the urinary tract, nearly 83% of *C. tropicalis* infections are associated with indwelling urinary catheters, and urinary isolates exhibit efficient adhesion to silicone surfaces in artificial urine, promoting persistence and complicating eradication of the infection [32,63,84].

The remarkable adhesive capacity of *C. tropicalis* has also been demonstrated on polystyrene surfaces, where this species adheres more efficiently than several other pathogenic *Candida* species, including multiple non-*albicans Candida* species. Adhesion to polystyrene is highly strain-dependent and varies according to the anatomical source of isolation [64,85,86]. Interestingly, adherence to polystyrene has been shown to exceed that observed for both HeLa and Vero epithelial cells, and a positive correlation has been reported between adhesion to abiotic surfaces and host cells [64]. These observations suggest that the molecular mechanisms governing the initial stages of adhesion are, at least in part, shared between biotic and abiotic substrates, thereby contributing to the remarkable ability of *C. tropicalis* to colonize medical devices and establish persistent infections [32].

Collectively, adhesion represents one of the major virulence determinants of *C. tropicalis*, serving as the foundation for subsequent pathogenic processes, including biofilm development, tissue invasion, and long-term persistence within the host. Although current evidence demonstrates that *C. tropicalis* shares numerous adhesion mechanisms with *C. albicans*, the specific functions of many adhesins and their associated regulatory networks remain poorly understood. Integrating phenotypic studies with comparative genomics and functional analyses will be essential to define the contribution of these determinants to *C. tropicalis* pathogenicity and to identify novel targets for antifungal intervention.

### 3.2. Biofilm Formation

Biofilm formation is one of the major virulence strategies employed by *C. tropicalis* and represents a direct consequence of its initial adhesion to host cells or abiotic surfaces [87]. Following attachment, fungal cells undergo a transition from a planktonic lifestyle to a highly organized sessile mode of growth, forming three-dimensional microbial communities embedded within a self-produced extracellular matrix [32,85]. This multicellular organization promotes long-term persistence, limits immune clearance, and markedly reduces susceptibility to antifungal agents, thereby contributing to chronic and device-associated infections [12,16,32].

Although *C. tropicalis* biofilms were historically considered comparable to those formed by *C. albicans*, accumulating evidence indicates that the two species differ substantially in biofilm architecture, developmental dynamics, and regulatory circuitry [88,89]. These findings suggest that, despite sharing several conserved molecular mechanisms, *C. tropicalis* has evolved species-specific strategies for biofilm development and maintenance that may contribute to its increasing clinical importance [89].

**Figure 3:**
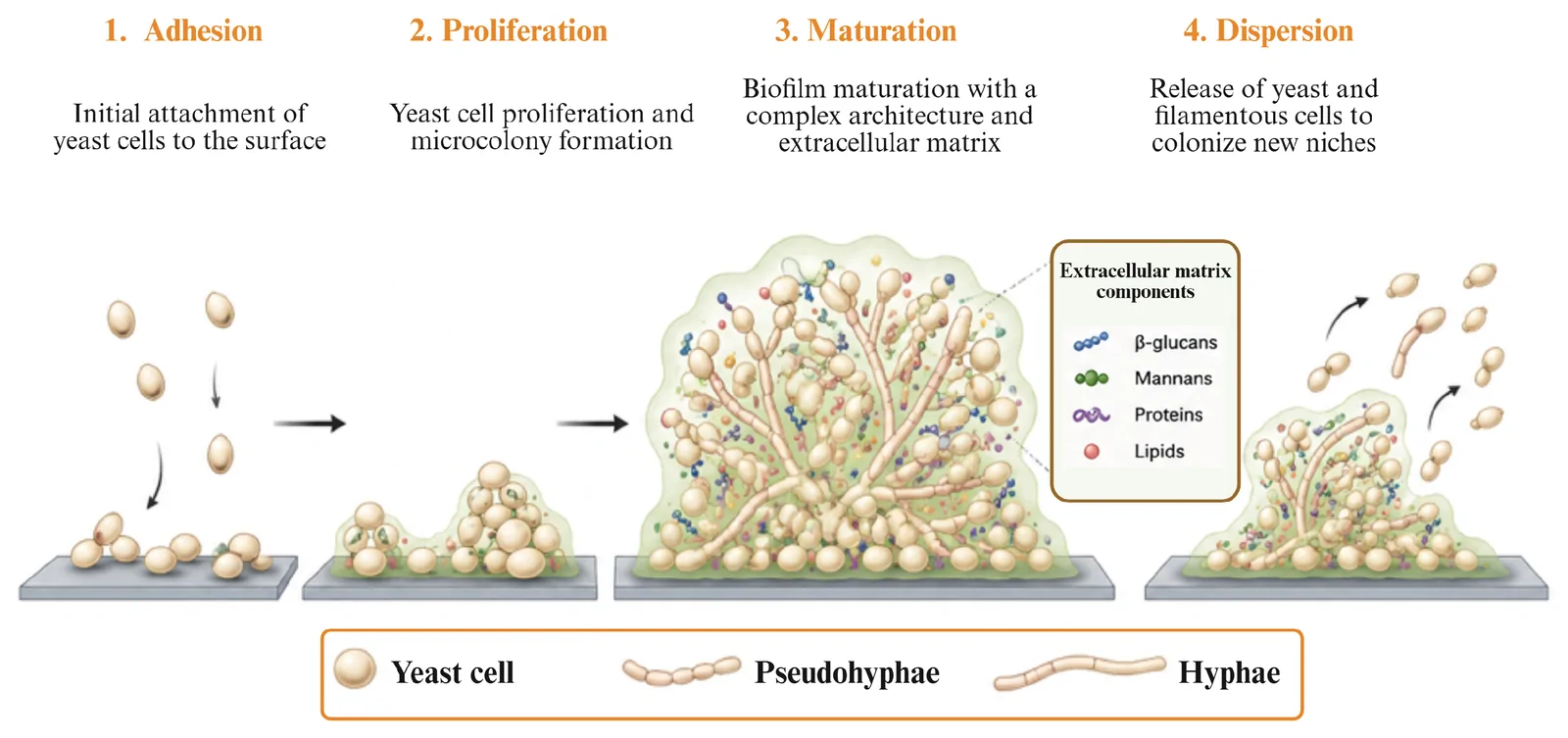
Schematic representation of biofilm development in *Candida tropicalis*. Biofilm formation occurs through four sequential stages: (1) adhesion, characterized by the initial attachment of yeast cells to the surface; (2) proliferation, involving yeast cell multiplication, microcolony formation, and the emergence of filamentous forms; (3) maturation, characterized by the development of a complex three-dimensional architecture composed of yeast cells, pseudohyphae, and hyphae embedded within an extracellular matrix containing β-glucans, mannans, proteins, and lipids; and (4) dispersion, during which yeast and filamentous cells are released from the mature biofilm and may colonize new niches. The schematic highlights the morphological diversity and progressive accumulation of extracellular matrix components associated with *C. tropicalis* biofilm development.

As in other *Candida* species, biofilm formation proceeds through four sequential stages: initial adhesion, cellular proliferation and microcolony formation, maturation of the three-dimensional biofilm structure, and dispersal of yeast cells capable of colonizing new niches (Figure 3) [90,91]. Throughout this process, extensive morphological remodeling occurs, involving transitions between yeast cells, pseudohyphae, and true hyphae, together with the progressive accumulation of an extracellular matrix composed primarily of β-glucans, mannans, proteins, lipids, and extracellular DNA (eDNA). In addition to providing structural stability, this hydrated matrix functions as a protective barrier against antifungal agents, immune cells, and other environmental stresses, thereby enhancing fungal survival and persistence [91].

Biofilm development in *C. tropicalis* is a highly dynamic and time-dependent process. Combined analyses of biomass accumulation, colony-forming unit counts, fluorescence microscopy, and scanning electron microscopy have demonstrated that viable cell numbers continue to increase for at least 96 h, whereas *C. albicans* typically reaches a stationary phase considerably earlier [89]. Interestingly, despite producing a smaller amount of extracellular matrix during late stages of development, *C. tropicalis* biofilms contain a larger number of viable cells than those formed by *C. albicans*, indicating fundamental differences in biofilm organization and physiology [89]. These observations suggest that biofilm-associated virulence depends not only on matrix production but also on growth dynamics and the metabolic activity of the embedded fungal cells [89,92].

Another distinctive feature of *C. tropicalis* is the pronounced heterogeneity in its biofilm-forming capacity. Studies involving both clinical and environmental isolates have revealed considerable strain-to-strain variation in biomass production, metabolic activity, and biofilm architecture [32,86,88]. Among environmental isolates recovered from coastal sand, approximately two-thirds were classified as strong biofilm producers, exhibiting levels comparable to those observed in candidemia-associated strains [12]. Biofilm formation is also strongly influenced by the surrounding microenvironment, including the nature of the substrate, culture medium composition, nutrient availability, pH, oxygen concentration, and the presence of metal ions [32,89]. Together, these findings highlight the remarkable phenotypic plasticity of *C. tropicalis* and help explain the variability reported among different experimental studies.

At the molecular level, biofilm development is controlled by a complex transcriptional regulatory network coordinating adhesion, morphological transitions, cell wall remodeling, and stress adaptation. In *C. albicans*, transcription factors such as Bcr1, Brg1, Efg1, Tec1, Ndt80, Rob1, and Csr1 constitute the core biofilm regulatory circuitry [93,94]. Although our BLASTp orthology analysis identified homologs of the principal biofilm regulators described in *C. albicans*, sequence conservation does not necessarily imply functional conservation [95]. Indeed, targeted mutagenesis studies in *C. tropicalis* have shown that gene deletion of *BCR1*, *BRG1*, *EFG1*, *TEC1*, or *NDT80* markedly impairs biofilm formation, whereas disruption of *ROB1* does not produce the dramatic phenotype observed in *C. albicans*. Likewise, expression of *CtROB1* fails to complement the *C. albicans rob1*Δ mutant, indicating that the regulatory function of this transcription factor has diverged between the two species. These findings demonstrate that, although the overall biofilm regulatory network is partially conserved, several of its components have undergone functional divergence during the evolution of *C. tropicalis* [95].

From a clinical perspective, *C. tropicalis* biofilms constitute an important reservoir of persistent cells and represent one of the major causes of therapeutic failure in device-associated infections [16,32]. The extracellular matrix restricts antifungal penetration, sequesters active compounds, and protects fungal cells from host immune defenses [16,96,97]. In addition, sessile cells undergo profound physiological adaptations, including reduced growth rates, metabolic reprogramming, increased expression of efflux pumps, and activation of stress-response pathways, collectively resulting in enhanced tolerance to azoles, polyenes, and, to a lesser extent, echinocandins [16,89,96]. Finally, dispersal of yeast cells from mature biofilms facilitates colonization of distant anatomical sites and contributes to recurrent episodes of candidemia [32,96].

Collectively, current evidence indicates that biofilm formation in *C. tropicalis* is a highly regulated and dynamic process integrating adhesion, morphological plasticity, cell wall remodeling, extracellular matrix production, and metabolic adaptation. Rather than representing a simple adhesive structure, biofilms function as multicellular survival communities that account for much of the persistence, antifungal tolerance, and clinical success of *C. tropicalis* as an opportunistic pathogen.

### 3.3. Morphological Plasticity and Dimorphism

The ability to undergo morphological transitions represents one of the most important virulence traits of *C. tropicalis*.

Filamentation in *C. tropicalis* can be induced by multiple environmental cues, including serum, physiological temperature (approximately 37 °C), pH fluctuations, elevated CO_2_ concentrations, nutrient availability, N-acetylglucosamine, and filamentation-inducing media such as Spider medium [98]. These signals are integrated through interconnected signaling pathways that regulate cell polarization, apical growth, cell wall remodeling, and the expression of morphogenesis-associated genes [12,30]. However, unlike *C. albicans*, the filamentation capacity of *C. tropicalis* exhibits substantial strain-to-strain variability and is strongly influenced by environmental conditions, underscoring the remarkable phenotypic plasticity of this species [12].

Morphological heterogeneity among *C. tropicalis* isolates has been extensively documented. Environmental isolates recovered from coastal sand develop distinct colony morphotypes when grown on Spider medium, including smooth, rough, and cottony colonies [12,24]. Cottony colonies are characterized by abundant hyphal and pseudohyphal growth; rough colonies display intermediate filamentation, whereas smooth colonies consist predominantly of yeast cells. These phenotypic differences reflect intrinsic variation in filamentation capacity and indicate that morphological transitions are governed by both the genetic background of the isolate and environmental conditions [24,32].

The transition between yeast and filamentous growth forms is closely integrated with other virulence determinants. During biofilm development, hyphae and pseudohyphae contribute to the three-dimensional architecture of the microbial community, reinforce structural stability, and enhance adhesion to both biotic and abiotic surfaces [89]. Filamentation also increases the invasive potential of *C. tropicalis*, as elongated filamentous structures promote directional growth through host tissues and facilitate penetration of epithelial barriers. Concurrently, morphological transitions are accompanied by dynamic remodeling of the cell wall and changes in the surface exposure of adhesins and other virulence-associated molecules, thereby modifying fungal interactions with host immune cells [16,32,89].

An additional mechanism linking filamentous growth to host cell damage is candidalysin, a cytolytic peptide toxin derived from the Ece1 protein [99]. In *C. tropicalis*, an *ECE1* ortholog containing a candidalysin-like region has been identified, and the corresponding peptide was shown to be biologically active [100,101]. The *C. tropicalis* candidalysin induces epithelial cell damage, Ca^2+^ influx, MAPK signaling, and IL-1α release, demonstrating its capacity to directly affect epithelial cells and activate damage-associated inflammatory responses [100]. Interestingly, the synthetic *C. tropicalis* candidalysin exhibited greater cytolytic activity and more rapid membrane permeabilization than its *C. albicans* counterpart. However, *C. tropicalis* itself caused less epithelial damage than *C. albicans*, indicating that the intrinsic activity of the peptide alone does not determine pathogenic outcome. Differences in filamentation, *ECE1* expression, peptide processing and secretion, and local toxin accumulation at the fungus–host interface may therefore modulate candidalysin-mediated damage among *Candida* species [100].

At the molecular level, morphogenesis is governed by a complex regulatory network coordinating cell polarization, cell wall remodeling, stress adaptation, and the expression of filamentation-associated genes. In *C. albicans*, transcriptional regulators such as Cph1, Hgc1, Nrg1, and Tup1 play central roles in controlling filamentous growth [102,103,104]. Consistent with these observations, our BLASTp orthology analysis identified homologs of Cph1, Hgc1, Nrg1, and Tup1 in *C. tropicalis* (Table 1), suggesting that much of the genetic machinery underlying morphological transitions is evolutionarily conserved between the two species. Nevertheless, sequence conservation does not necessarily imply functional equivalence, and the precise contribution of these regulators to morphogenesis in *C. tropicalis* remains to be experimentally established [105].

Several studies have further demonstrated that cell wall integrity is essential for filamentation in *C. tropicalis* [39]. In particular, disruption of *PMT2*, which encodes a protein *O*-mannosyltransferase involved in the *O*-linked mannosylation of cell wall proteins, results in abnormal cellular morphology, significantly impaired hyphal and biofilm formation, and reduced interaction with host cells. These findings demonstrate that proper glycosylation of cell wall proteins directly contributes to the regulation of dimorphism and establishes a functional link between cell wall organization, morphogenesis, and virulence in *C. tropicalis* [39].

Beyond the classical yeast-to-hypha transition, *C. tropicalis* possesses additional mechanisms of morphological plasticity through phenotypic switching. This species undergoes a tri-stable white-gray-opaque switching program that is primarily regulated by the transcription factor Wor1, with each phenotypic state displaying distinct morphological, metabolic, mating, and virulence characteristics [12,30]. Transitions between these phenotypic states enhance fungal adaptation to different ecological niches and host environments, representing an additional regulatory layer that complements the classical dimorphic program and further expands the pathogenic potential of *C. tropicalis* [29,30].

Collectively, morphological plasticity represents a fundamental adaptive strategy in *C. tropicalis*, integrating environmental sensing, genetic regulation, cell wall remodeling, and physiological adaptation to promote host colonization, tissue invasion, and long-term persistence. The intimate relationship between morphogenesis and other virulence determinants highlights phenotypic plasticity as one of the principal factors underlying the remarkable pathogenic success of this opportunistic fungal pathogen.

### 3.4. Immune Evasion

**Figure 4:**
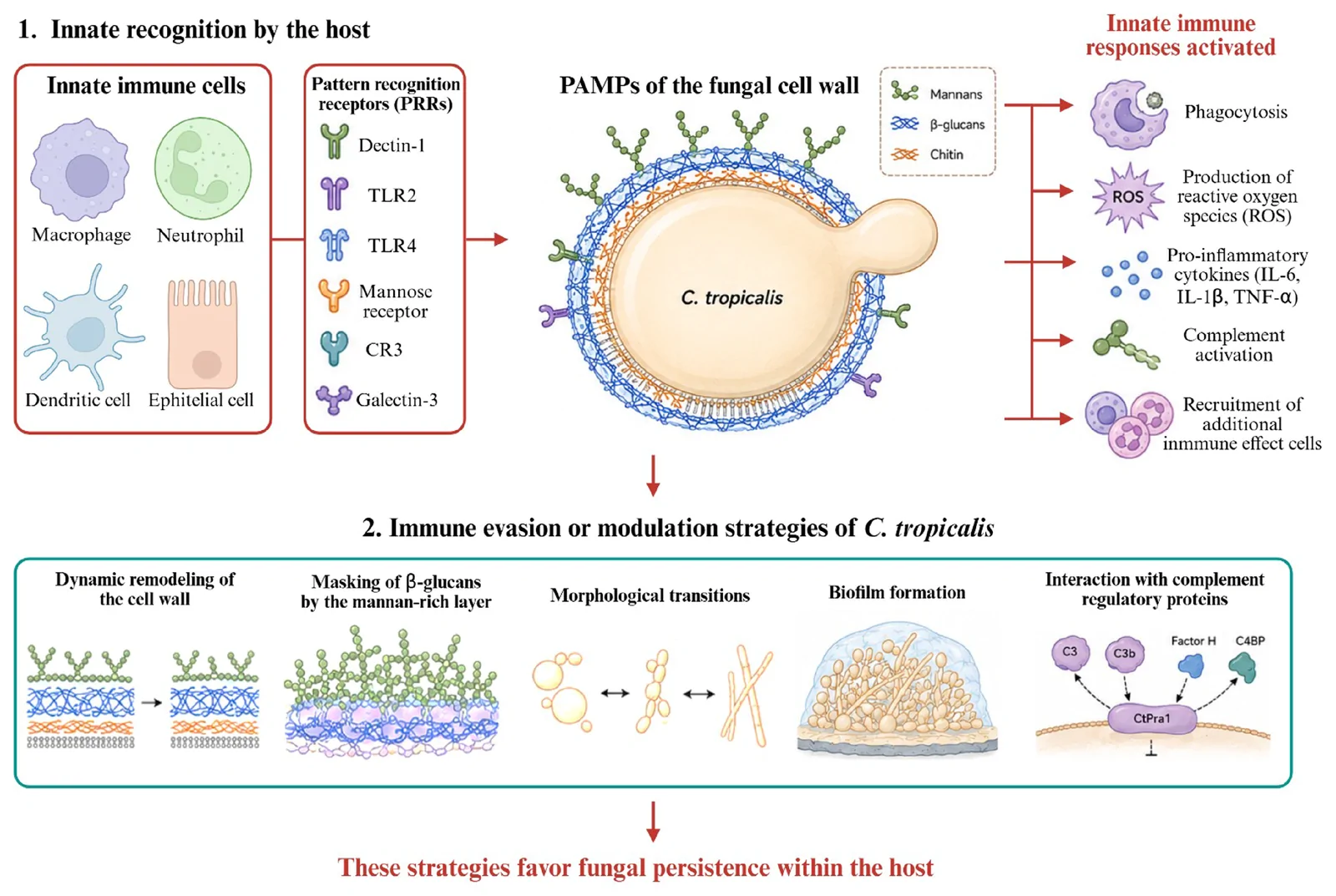
Innate immune recognition and immune evasion strategies of *Candida tropicalis*. Following host colonization, *C. tropicalis* is recognized by innate immune cells, including macrophages, neutrophils, dendritic cells, and epithelial cells, through pattern recognition receptors (PRRs) that detect fungal cell wall-associated pathogen-associated molecular patterns (PAMPs), such as mannans, β-glucans, and chitin. This recognition triggers innate immune responses, including phagocytosis, reactive oxygen species (ROS) production, pro-inflammatory cytokine secretion, complement activation, and recruitment of additional immune effector cells. In parallel, *C. tropicalis* employs multiple strategies to evade or modulate host immunity, including dynamic cell wall remodeling, masking of β-glucans by the outer mannan-rich layer, morphological transitions, biofilm formation, and interactions with complement regulatory proteins. Collectively, these mechanisms reduce or modify immune recognition and favor fungal persistence within the host.

The interaction between *C. tropicalis* and the host immune system is a dynamic process that largely determines the establishment and progression of infection. Following initial colonization, fungal cells are primarily recognized by the innate immune system through pattern recognition receptors (PRRs) expressed on macrophages, neutrophils, dendritic cells, and epithelial cells (Figure 4) [35,106]. These receptors detect conserved fungal cell wall components, known as pathogen-associated molecular patterns (PAMPs), including β-glucans, mannans, and chitin. Recognition of these molecules triggers phagocytosis, reactive oxygen species (ROS) production, secretion of pro-inflammatory cytokines, complement activation, and recruitment of additional immune effector cells [38,107].

Nevertheless, *C. tropicalis* has evolved multiple strategies to evade or modulate these host defenses. These include dynamic remodeling of the cell wall, masking of β-glucans by the outer mannan-rich layer, morphological transitions, biofilm formation, and interactions with complement regulatory proteins (Figure 4) [108]. Acting in concert with other virulence determinants, these mechanisms promote fungal persistence within diverse host niches and contribute to the establishment of invasive disease [33].

One of the best-characterized immune evasion mechanisms in *C. tropicalis* involves dynamic remodeling of the fungal cell wall [35,44]. The spatial organization of structural polysaccharides determines the accessibility of major PAMPs to host immune receptors. Under basal conditions, much of the β-1,3-glucan and chitin remain partially concealed beneath the outer mannan layer, thereby limiting recognition by receptors such as Dectin-1 and modulating downstream inflammatory responses [35]. Functional analyses of mutants defective in *N*- and *O*-linked mannan biosynthesis demonstrated that alterations in cell wall glycosylation increase the surface exposure of β-glucans and chitin, modify recognition by human macrophages, and significantly alter the production of both pro- and anti-inflammatory cytokines, including TNF-α, IL-6, and IL-10 [35]. These studies further revealed that immune recognition of *C. tropicalis* depends on the coordinated activity of multiple PRRs, including Dectin-1, TLR2, TLR4, the mannose receptor, CR3, and galectin-3, whose relative contributions vary according to the composition and organization of the fungal cell wall [35].

More recent evidence indicates that cell wall remodeling also represents an adaptive mechanism linked to antifungal resistance [44]. Integrated transcriptomic, metabolomic, and cell wall composition analyses demonstrated that fluconazole-resistant isolates exhibit reduced surface exposure of β-1,3-glucan together with altered expression of genes involved in cell wall biosynthesis and remodeling. Consequently, these isolates display diminished recognition by Dectin-1 and an enhanced capacity to evade innate immune surveillance. In addition, resistant strains undergo profound metabolic reprogramming, including alterations in lipid metabolism and increased ergosterol content, suggesting that antifungal resistance and immune evasion are tightly interconnected processes during host adaptation. Collectively, these findings demonstrate that cell wall remodeling is not merely a physiological adaptation but rather a central mechanism by which *C. tropicalis* regulates its immunogenicity and promotes persistence during infection [35,44].

The complement system constitutes another major barrier against *Candida* infection by promoting opsonization, phagocyte recruitment, and fungal clearance [107]. However, recent studies have demonstrated that *C. tropicalis* possesses specific mechanisms to interfere with complement activation. The surface protein CtPra1 (pH-regulated antigen 1) binds C3 and C3b while simultaneously recruiting the complement regulators factor H and C4b-binding protein (C4BP). As a consequence, activation of the classical, alternative, and lectin pathways is attenuated, resulting in reduced C3b deposition on the fungal surface and enhanced resistance to innate immune defenses [33,109]. Furthermore, the secreted aspartyl protease Sapt1 has been reported to degrade or interfere with molecules involved in lectin pathway activation, including mannose-binding lectin (MBL) and collectin-11, while also impairing interactions with the dendritic cell receptor DC-SIGN, thereby further reducing immune recognition of the fungus [109].

Biofilm formation represents an additional mechanism of immune evasion. The extracellular matrix restricts access of neutrophils, macrophages, and soluble immune effectors while simultaneously limiting antifungal penetration into the deeper layers of the biofilm [97]. In parallel, sessile cells undergo extensive physiological and metabolic adaptations that enhance stress tolerance and promote long-term persistence during infection. Together, these features establish biofilms as important reservoirs of persistent cells and largely explain the therapeutic failure frequently observed in device-associated infections [16,32,97].

Beyond avoiding immune recognition, *C. tropicalis* is also capable of actively modulating host inflammatory responses [106]. In a murine model of systemic candidiasis, infection induced profound leukocyte redistribution characterized by a reduction in circulating immune cells and marked recruitment to the kidneys, the principal target organ during disseminated candidiasis. This inflammatory response was accompanied by extensive leukocyte infiltration and tissue damage, suggesting that disease progression depends not only on fungal immune evasion but also on the capacity of *C. tropicalis* to influence leukocyte trafficking and the magnitude of the host inflammatory response. These findings highlight immunopathology as an important component of *C. tropicalis* infections, where the balance between pathogen clearance and immune-mediated tissue damage may ultimately determine disease outcome [106].

From a comparative genomics perspective, our BLASTp orthology analysis identified putative orthologs of *PRA1*, *HGT1*, and *MSB2* in *C. tropicalis* (Table 1), three genes that contribute to distinct immune evasion mechanisms in *C. albicans*. Pra1 is among the best-characterized factors, as its role in recruiting negative regulators of the complement cascade has been experimentally demonstrated in both *C. albicans* and *C. tropicalis* [109]. In contrast, Hgt1 functions in *C. albicans* not only as a glucose transporter but also as a surface protein capable of recruiting factor H, thereby suppressing complement activation [110]. Likewise, Msb2 is a transmembrane mucin involved in cell wall integrity, Cek1 signaling, stress adaptation, and protection against antimicrobial peptides. Although the roles of Hgt1 and Msb2 have not yet been experimentally validated in *C. tropicalis*, the identification of their putative orthologs suggests that similar immune evasion strategies may be conserved in this species and warrant future functional investigation [33,109].

Collectively, immune evasion in *C. tropicalis* results from the integration of structural, morphological, and molecular mechanisms. Cell wall remodeling, biofilm formation, complement interference, and modulation of host inflammatory responses collectively reduce immune recognition and promote fungal persistence within the host. The identification of putative orthologs of *PRA1*, *HGT1*, and *MSB2* further supports the concept that *C. tropicalis* shares several immune evasion strategies with other pathogenic *Candida* species, although functional characterization of these genes remains an important challenge for future research.

### 3.5. Extracellular Hydrolytic Enzymes

The secretion of extracellular hydrolytic enzymes represents one of the major virulence strategies employed by *C. tropicalis* during host colonization and infection [111]. These enzymes contribute to the degradation of host structural components, nutrient acquisition, tissue invasion, and modulation of host immune responses. Among the best-characterized families are the secreted aspartyl proteinases (Saps) and phospholipases, both of which are widely produced by clinical isolates of *C. tropicalis* and are generally associated with increased pathogenic potential [33].

Saps hydrolyze a broad range of host proteins, including albumin, collagen, laminin, fibronectin, immunoglobulins, and complement components. Through their proteolytic activity, these enzymes facilitate tissue penetration, provide amino acids as carbon and nitrogen sources, and disrupt both physical and immunological barriers [112]. In *C. tropicalis*, at least four secreted aspartyl proteinase genes, *SAPT1–SAPT4*, have been described, with Sapt1 representing the best biochemically characterized member of this family. Sapt1 was purified from culture supernatants and identified as the predominant aspartyl proteinase secreted when bovine serum albumin was provided as the sole nitrogen source, supporting a role for extracellular proteolysis in nutrient acquisition [99]. Importantly, expression of individual *SAPT* genes appears to be highly dependent on environmental conditions and the infection context, indicating that the contribution of each proteinase may vary considerably during host colonization and disease. In addition, members of the Sapt family have been implicated in interactions with host immune components, further suggesting that extracellular proteolysis may contribute not only to nutrient acquisition and tissue damage but also to modulation of host–fungus interactions [33,109,113].

Several studies have demonstrated considerable variability in *SAP* activity among *C. tropicalis* isolates, with proteolytic activity generally being higher in strains recovered from invasive infections than in commensal or environmental isolates. Moreover, increased *SAP* production is frequently associated with enhanced biofilm formation, filamentation, and tissue invasion, highlighting the close functional relationship between extracellular proteolysis and other virulence determinants [33]. Recent phenotypic analyses of clinical isolates further confirmed that protease activity is among the most prevalent virulence-associated traits of *C. tropicalis*, although its expression varies markedly among strains [56,114].

Phospholipases constitute another important family of hydrolytic enzymes involved in *C. tropicalis* pathogenicity. These enzymes catalyze the hydrolysis of phospholipids present in host cell membranes, thereby compromising membrane integrity and facilitating cellular damage, tissue invasion, and fungal dissemination [12]. In addition, phospholipid degradation products may alter the local inflammatory microenvironment and promote colonization of new anatomical niches. Multiple studies have demonstrated phospholipase activity in *C. tropicalis*, although considerable variability exists among clinical isolates, with invasive strains generally exhibiting greater enzymatic activity than colonizing or environmental isolates [12,33,114].

Production of both proteinases and phospholipases is highly heterogeneous among *C. tropicalis* isolates. Phenotypic studies have revealed substantial differences in the intensity of enzymatic activity, suggesting that expression of these virulence factors is influenced by strain-specific characteristics as well as environmental conditions encountered during infection. Interestingly, isolates exhibiting high extracellular enzymatic activity frequently display enhanced adhesion, filamentation, and biofilm-forming capacity, indicating that these virulence determinants are likely co-regulated during host colonization and disease progression [12,114].

From a comparative genomics perspective, our BLASTp orthology analysis identified putative orthologs of the major secreted aspartyl proteinase and phospholipase families previously characterized in *C. albicans* (Table 1). Together with the available phenotypic evidence, these findings highlight the extensive repertoire of hydrolytic enzymes in *C. tropicalis* potentially involved in tissue degradation, nutrient acquisition, remodeling of the host microenvironment, and immune evasion. Nevertheless, available phenotypic studies consistently demonstrate pronounced variability in the production of both proteinases and phospholipases among clinical isolates, indicating that transcriptional regulation, environmental conditions, fungal morphology, and host-derived signals play major roles in determining the expression and activity of these enzymes [114].

Collectively, extracellular hydrolytic enzymes constitute essential virulence determinants that enable *C. tropicalis* to invade host tissues, exploit host-derived nutrients, modulate immune responses, and establish persistent infections. Although comparative genomic analyses indicate that the principal enzymatic families are evolutionarily conserved between *C. tropicalis* and *C. albicans*, functional characterization of many of these enzymes remains incomplete. Future studies integrating comparative genomics, transcriptomics, proteomics, and functional genetics will be essential to clarify how extracellular hydrolytic enzymes contribute to the pathogenic success of *C. tropicalis* and to evaluate their potential as targets for novel antifungal strategies.

### 3.6. Thermotolerance

The ability to grow and survive at host body temperature is a fundamental prerequisite for the pathogenicity of opportunistic fungi [115,116]. In *C. tropicalis*, thermotolerance enables colonization of human tissues and facilitates the transition from environmental or commensal niches to a pathogenic lifestyle. This trait becomes particularly important during invasive candidiasis, where the fungus must adapt not only to physiological temperature (37 °C) but also to febrile conditions that may exceed 39 °C [117]. Successful adaptation to thermal stress requires extensive physiological and molecular reprogramming that promotes fungal survival under adverse host conditions.

The response to heat stress is primarily mediated by heat shock proteins (Hsps), a conserved family of molecular chaperones that preserve protein folding, prevent aggregation of denatured proteins, and maintain cellular proteostasis [118]. Although the functional characterization of Hsps in *C. tropicalis* remains considerably less advanced than in *C. albicans*, comparative genomic analyses indicate the conservation of several *HSP* gene families, including *HSP60*, *HSP70*, *HSP90*, and *HSP104*, suggesting that similar thermoadaptive mechanisms are likely to operate in this species. In *C. albicans*, Hsp90 represents a central regulator of cellular stress responses by stabilizing multiple client proteins involved in cell wall integrity, morphogenesis, signal transduction, and the development of antifungal tolerance and resistance [119,120]. Although these mechanisms have not yet been fully characterized in *C. tropicalis*, the strong evolutionary conservation of *HSP* genes suggests that they may perform comparable biological functions [118].

Thermotolerance should not be regarded as an isolated virulence trait but rather as part of an integrated physiological adaptation network closely connected with other pathogenicity determinants [33]. Elevated temperature induces extensive cell wall remodeling, modifies the exposure of PAMPs, promotes transitions between yeast and filamentous morphologies, and may enhance biofilm development [120]. In addition, the heat stress response is tightly linked to oxidative stress adaptation, cellular homeostasis, and metabolic reprogramming, enabling *C. tropicalis* to maintain viability under the hostile conditions encountered during infection [44].

Recent studies further indicate that the heat stress response forms part of a broader regulatory network integrating environmental sensing, nutrient availability, and host adaptation. Consequently, thermotolerance is best considered a component of the remarkable physiological plasticity of *C. tropicalis* rather than an independent virulence factor [12,24]. The close interplay between thermal adaptation, cell wall remodeling, filamentation, biofilm formation, immune evasion, and antifungal resistance underscores the central role of thermotolerance in promoting fungal persistence during infection [44]. Nevertheless, the molecular mechanisms coordinating these interconnected processes remain poorly understood and represent an important area for future investigation.

From a comparative genomics perspective, our BLASTp orthology analysis identified putative orthologs of *HSP60* and *HSP104* in *C. tropicalis* (Table 1), two highly conserved molecular chaperones involved in the response to thermal stress. These orthologs displayed high amino acid identity with their *C. albicans* counterparts (97% and 96%, respectively), supporting the conservation of fundamental thermoadaptive mechanisms between both species. In contrast, no putative ortholog of *SSA1* was identified using the criteria applied in this study. Given that the Hsp70 protein family comprises multiple closely related paralogs and that gene annotation differs among *Candida* species, this result should not be interpreted as evidence for the absence of Ssa1-like functions in *C. tropicalis*. Instead, it likely reflects divergence in Hsp70 family organization or inherent limitations of sequence-based orthology analyses [118,121]. Additional phylogenetic, transcriptomic, and functional studies will be required to determine whether these functions are carried out by alternative Hsp70 homologs in *C. tropicalis*.

Finally, thermotolerance represents a fundamental component of the physiological plasticity and adaptive capacity of *C. tropicalis*. Rather than acting as an independent virulence determinant, adaptation to thermal stress is integrated with cell wall remodeling, morphogenesis, biofilm development, immune evasion, and antifungal resistance, collectively promoting fungal survival and persistence within the host. Continued advances in comparative genomics, transcriptomics, proteomics, and functional genetics will be instrumental in elucidating the molecular networks underlying thermal adaptation and defining the precise contribution of heat shock proteins to the pathogenesis of *C. tropicalis*.

## 4. Candidiasis Caused by Candida Tropicalis

*C. tropicalis* is an opportunistic pathogen capable of causing a broad spectrum of clinical manifestations, ranging from superficial mucocutaneous infections to invasive candidiasis and candidemia [12]. Although this species may colonize the skin and mucosal surfaces of the gastrointestinal, genitourinary, and respiratory tracts, disruption of host barriers or impairment of immune responses can favor its transition from commensal colonizer to invasive pathogen. This transition is particularly relevant in hospitalized patients, where prolonged exposure to broad-spectrum antibiotics, central venous catheters, parenteral nutrition, abdominal surgery, intensive care unit admission, neutropenia, hematological malignancies, chemotherapy, and hematopoietic stem cell transplantation constitute major predisposing factors for invasive disease [10].

Clinically, *C. tropicalis* has been associated with oral and vulvovaginal candidiasis, cutaneous infections, onychomycosis, urinary tract infections, peritonitis, candidemia, and disseminated candidiasis [10]. However, its greatest clinical impact is observed in bloodstream and deep-seated infections. In recent decades, the epidemiology of invasive candidiasis has shifted from infections predominantly caused by *C. albicans* toward a growing participation of non-*albicans Candida* species [122,123]. Within this group, *C. tropicalis* has emerged as one of the most frequent causes of candidemia, particularly in Asia, Latin America, and other tropical or subtropical regions, where it has been reported as the first or second most common cause of *Candida* bloodstream infections in some studies [9,10].

A systematic review performed to inform the World Health Organization (WHO) fungal priority pathogens list included 30 studies comprising 436 patients from 25 countries and highlighted the global clinical impact of invasive *C. tropicalis* infections. In this analysis, all-cause mortality associated with invasive *C. tropicalis* infections reached 55–60%, whereas 30-day mortality in candidemia ranged from 32% to 52% [10]. In pediatric patients with invasive *C. tropicalis* infections, overall mortality was reported between 26% and 40% [10]. These values emphasize the high lethality of infections caused by this species, especially when diagnosis or antifungal treatment is delayed.

Among the populations at highest risk, patients with hematological malignancies and prolonged neutropenia represent one of the most vulnerable groups. Invasive *C. tropicalis* infections have been strongly associated with leukemia, with an odds ratio of 4.77 compared with infections caused by other *Candida* species, while chronic lung disease has also been identified as a significant risk factor (OR = 2.62) [10,124]. In hematology wards, *C. tropicalis* bloodstream infection is frequently linked to mucosal barrier injury, chemotherapy-induced neutropenia, central venous catheters, previous antifungal exposure, and prolonged hospitalization [124].

A retrospective study of 26 patients with hematological diseases and *C. tropicalis* bloodstream infection reported that 88.5% had hematological malignancies and all patients presented neutropenia. In this cohort, total mortality was 42.3%, while attributable mortality reached 34.6% [105]. Septic shock was identified as an independent risk factor for attributable death, and delays in antifungal treatment were associated with poor prognosis. Specifically, a time from fever onset to antifungal therapy of ≥5 days and a time from neutropenia to antifungal therapy of ≥10 days were associated with increased mortality, underscoring the importance of early clinical suspicion and prompt antifungal intervention in high-risk patients [105].

The clinical presentation of candidemia caused by *C. tropicalis* is often nonspecific and may include persistent fever, chills, hypotension, septic shock, respiratory symptoms, gastrointestinal manifestations, skin or mucosal lesions, and laboratory evidence of systemic inflammation [125]. Because these signs overlap with bacterial sepsis, early diagnosis remains challenging. Blood culture continues to be the diagnostic standard for candidemia, but its sensitivity is limited, and species identification may be delayed. This diagnostic delay is especially problematic in neutropenic or critically ill patients, in whom disease progression can be rapid, and mortality is high [105,124].

In addition to bloodstream infection, *C. tropicalis* can cause disseminated candidiasis with involvement of deep organs such as the liver, spleen, kidney, lung, retina, skin, and central nervous system [105,126]. Hepatosplenic candidiasis is particularly relevant in patients with hematological malignancies and prolonged neutropenia, often appearing during neutrophil recovery after chemotherapy. Cutaneous lesions may also occur as a manifestation of hematogenous dissemination and can provide an important clinical clue in patients with persistent fever and suspected invasive fungal infection [105].

Another clinically relevant aspect is the ability of *C. tropicalis* to form biofilms on biotic and abiotic surfaces, including central venous catheters and other medical devices. Biofilm formation contributes to persistence, reduced antifungal susceptibility, immune evasion, and therapeutic failure [12,127]. Several studies have suggested that strong biofilm-forming isolates are associated with more severe clinical outcomes and may contribute to the high mortality observed in candidemia. Therefore, removal or replacement of infected intravascular catheters, when feasible, is an important component of clinical management [127,128,129].

Mixed candidemia should also be considered in immunocompromised patients. Although relatively uncommon, mixed infections have been reported in 2–9.3% of candidemia cases and may involve *C. tropicalis* together with other clinically relevant yeasts, including *C. albicans*, *C. glabrata* (currently *Nakaseomyces glabrata*), *C. parapsilosis*, and emerging multidrug-resistant species such as *Candida auris* (currently *Candidozyma auris*) [125]. The use of chromogenic media and molecular identification methods can improve the detection of mixed candidemia, which may otherwise remain unrecognized by conventional culture-based approaches. This is clinically important because co-infecting species may display markedly different antifungal susceptibility profiles, potentially affecting treatment response.

The management of candidiasis caused by *C. tropicalis* is further complicated by increasing antifungal resistance, particularly to azoles. Resistance to fluconazole and other triazoles varies geographically, but rates as high as 36–42% for fluconazole and even higher non-wild-type rates for some triazoles have been reported in selected studies. In contrast, resistance to echinocandins, amphotericin B, and flucytosine remains generally low, although continuous surveillance is essential [10,130,131]. In hematological patients, one study reported intermediate or resistant phenotypes to fluconazole, itraconazole, and voriconazole in 41.7%, 50%, and 41.7% of *C. tropicalis* isolates, respectively, while all isolates were susceptible to amphotericin B and flucytosine [105].

Taken together, candidiasis caused by *C. tropicalis* represents a major clinical concern due to its increasing prevalence among non-*albicans Candida* infections, its association with highly vulnerable patient populations, its ability to disseminate systemically, and its high mortality rates [10]. The combination of virulence traits, biofilm formation, diagnostic challenges, and emerging azole resistance highlights the need for early species-level identification, antifungal susceptibility testing, appropriate source control, and continued epidemiological surveillance.

## 5. Identification and Diagnosis

Accurate identification of *C. tropicalis* is essential for appropriate clinical management, epidemiological surveillance, and antifungal susceptibility assessment. Although this species exhibits characteristic phenotypic features that facilitate its differentiation from several other members of the genus, the use of molecular and proteomic approaches has significantly improved the accuracy and speed of identification in clinical laboratories [12].

### 5.1. Microbiological and Biochemical Identification

Conventional culture-based methods remain the initial approach for the identification of *C. tropicalis*. On Sabouraud dextrose agar, colonies typically appear white to cream-colored, with a creamy texture and a smooth surface that may become slightly wrinkled with prolonged incubation [15]. On CHROMagar *Candida*, *C. tropicalis* generally produces metallic blue to dark blue colonies after incubation, facilitating its presumptive differentiation from other clinically relevant *Candida* species. Similar chromogenic media, including *Candida* ID2, CandiSelect™ 4, and Brilliance *Candida* Agar, have also been successfully employed for species differentiation based on colony coloration generated by species-specific enzymatic activities [12,132].

Microscopic examination of cultures grown on cornmeal agar supplemented with Tween 80 reveals spherical to ovoid blastoconidia, pseudohyphae, and true hyphae, morphological characteristics commonly associated with this species [16]. In addition, *C. tropicalis* is capable of fermenting and assimilating several carbohydrates, including galactose, sucrose, maltose, and trehalose, properties that have historically been exploited for species identification through biochemical assays [12,15].

Several commercial biochemical identification systems have demonstrated high accuracy for the identification of *C. tropicalis*. Among these, API ID 32C, API 20C AUX, AuxaColor™, VITEK 2, BD Phoenix™, and Candifast have been widely evaluated and generally show excellent performance when identifying this species, although differences in overall accuracy have been reported among platforms [133,134,135].

### 5.2. Proteomic and Molecular Identification

Matrix-assisted laser desorption ionization–time of flight mass spectrometry (MALDI-TOF MS) has emerged as one of the most reliable methods for yeast identification and is currently implemented in many clinical microbiology laboratories. Studies evaluating both the VITEK MS and MALDI Biotyper platforms have reported identification accuracies approaching 100% for *C. tropicalis*, highlighting the robustness and rapidity of this technology [135,136,137,138,139].

Fluorescence in situ hybridization using peptide nucleic acid probes (PNA-FISH) has also been successfully employed for the rapid identification of *Candida* species directly from clinical samples, with reported sensitivities and specificities ranging from 96% to 100% for *C. tropicalis* [140,141,142,143].

DNA-based methods remain the gold standard for definitive species identification. These approaches commonly target ribosomal DNA regions, including the internal transcribed spacer (*ITS1* and *ITS2*) regions and the D1/D2 domains of the 26S rRNA gene, which contain sufficient sequence variability to discriminate among closely related yeast species [139,144]. Beyond species identification, molecular typing methods such as microsatellite analysis and random amplified polymorphic DNA (RAPD)-PCR have proven useful for investigating genetic diversity, population structure, and epidemiological relationships among *C. tropicalis* isolates [145,146].

Recent advances in molecular diagnostics have further improved the detection and identification of *C. tropicalis*. Real-time PCR assays and multiplex PCR platforms enable rapid and sensitive identification directly from clinical specimens, reducing the time required for diagnosis compared with conventional culture-based methods [147]. Furthermore, next-generation sequencing (NGS) technologies have emerged as valuable tools for the comprehensive characterization of fungal communities and the detection of mixed infections, particularly in cases where conventional methods may fail to identify uncommon or closely related species [148]. Although these approaches remain relatively expensive and are not yet routinely implemented in many clinical laboratories, their increasing accessibility is expected to enhance the diagnosis, epidemiological surveillance, and molecular characterization of *C. tropicalis* infections in the future.

## 6. Treatment

The treatment of *C. tropicalis* infections remains challenging due to factors such as delayed diagnosis, the limited number of antifungal drug classes available for clinical use, host-related factors, and the increasing emergence of antifungal resistance. The choice of therapy depends on the site of infection, disease severity, underlying patient conditions, and local antifungal susceptibility patterns. In cases of invasive candidiasis and candidemia, current clinical guidelines generally recommend echinocandins as first-line therapy because of their broad activity against most *Candida* species and their favorable safety profile [9].

Echinocandins, including caspofungin, micafungin, and anidulafungin, inhibit β-1,3-glucan synthesis, thereby compromising fungal cell wall integrity. These agents exhibit potent activity against most *C. tropicalis* isolates and are currently considered the preferred treatment for invasive infections, particularly in critically ill or immunocompromised patients [10,149].

Amphotericin B remains an effective therapeutic option for severe candidiasis and invasive fungal infections. This polyene exerts its antifungal activity by binding to ergosterol in the fungal plasma membrane, leading to pore formation and cell death. Although resistance to amphotericin B remains relatively uncommon in *C. tropicalis*, several surveillance studies have reported gradual increases in minimum inhibitory concentration (MIC) values among clinical isolates, highlighting the importance of continuous susceptibility monitoring [16,150].

Among azole antifungals, fluconazole has historically been one of the most widely used agents for the treatment of candidiasis due to its favorable pharmacokinetic properties and oral availability. However, increasing rates of fluconazole resistance have been reported worldwide in *C. tropicalis*, particularly among isolates recovered from invasive infections. Other triazoles, including itraconazole, voriconazole, and posaconazole, generally exhibit greater in vitro activity against *C. tropicalis*, with lower MIC values reported for many clinical isolates [150]. Nevertheless, cross-resistance among azoles has been increasingly documented, particularly in regions with extensive antifungal exposure.

The emergence of antifungal resistance represents one of the greatest challenges in the management of *C. tropicalis* infections. Resistance mechanisms include alterations in ergosterol biosynthesis pathways, overexpression of efflux pumps, mutations in drug target genes, and adaptive responses associated with biofilm formation. Consequently, antifungal susceptibility testing has become an important component of clinical management, particularly in invasive infections and in regions where azole-resistant *C. tropicalis* isolates are prevalent [9,10].

## 7. Conclusions

*C. tropicalis* has become one of the most clinically relevant non-*albicans Candida* species because of its increasing global prevalence, strong association with invasive disease in highly vulnerable patients, substantial mortality, and growing resistance to commonly used antifungal agents. Its pathogenic success cannot be attributed to a single virulence determinant but rather to the coordinated interaction of multiple adaptive traits, including cellular adhesion, biofilm formation, morphological plasticity, extracellular hydrolytic enzyme production, thermotolerance, cell wall remodeling, immune evasion, and genomic plasticity. Together, these mechanisms enable the fungus to colonize diverse host niches, withstand environmental and immune pressures, persist on medical devices and within tissues, and disseminate during invasive infection.

The available evidence further indicates that virulence and antifungal resistance are closely interconnected. Genomic variation, loss of heterozygosity, aneuploidy, gene amplification, transcriptional reprogramming, and cell wall remodeling can simultaneously alter fungal fitness, drug susceptibility, stress tolerance, and immune recognition. This biological interdependence may help explain the emergence and expansion of resistant *C. tropicalis* lineages and highlights the limitations of considering resistance, virulence, and host adaptation as independent phenomena.

The comparative BLASTp analysis included in this review identified putative *C. tropicalis* orthologs of numerous *C. albicans* proteins involved in adhesion, biofilm development, morphogenesis, immune evasion, hydrolytic enzyme activity, and thermal adaptation. These findings support the evolutionary conservation of a substantial portion of the molecular machinery associated with pathogenicity. Nevertheless, sequence similarity alone does not demonstrate functional equivalence, and the biological contribution of many of these candidates remains to be established through targeted mutagenesis, transcriptional profiling, proteomic analyses, and physiologically relevant infection models.

Despite recent advances, major knowledge gaps remain. Future studies should prioritize the functional characterization of species-specific virulence networks, the identification of biomarkers associated with invasive disease and therapeutic failure, and the clarification of how clinical, environmental, and animal reservoirs contribute to the emergence and dissemination of resistant strains. The integration of genomic surveillance with standardized susceptibility testing, biofilm assessment, host–pathogen interaction studies, and clinical metadata will be essential to improve risk stratification and guide antifungal therapy. Ultimately, a more comprehensive understanding of the molecular and ecological mechanisms underlying *C. tropicalis* pathogenicity will be required to strengthen diagnosis, optimize treatment, and identify new therapeutic or preventive targets against this increasingly important opportunistic pathogen.

## Figures and Tables

**Table 1:** Putative virulence factors identified in *Candida tropicalis* by BLASTp analysis.

Virulence Factor	*Candida albicans* Protein	*Candida tropicalis* Protein *	E-Value **	Similarity (%) **
Adhesins	Als1, Als3	CTRG_02293	0	74
	Als2, Als4	No found	-	-
	Als5	CTRG_01028	0	69
	Eap1/Hwp1	CTRG_00477	1 × 10^−17^	46
	Ecm33	JTP64_005625	0	85
	Iff4	CTRG_05198	9 × 10^−150^	78
	Int1	CTRG_06016	0	71
	Mp65	CTRG_02093	1 × 10^−177^	81
	Phr1	CTRG_03942	0	86
Biofilms	Bcr1	CTRG_00608	0	64
	Brg1	CTRG_04523	1 × 10^−49^	95
	Efg1	Efg1	2 × 10^−139^	62
	Hsp90	K6H09_003792	0	97
	Ndt80	CTRG_01069	0	69
	Rob1	JTP64_004426	0	59
	Csr1	K4I79_001648	0	71
Dimorphism	Cph1	CTRG_04159	0	73
	Hgc1	K6H11_001669	3 × 10^−21^	46
	Nrg1	K6H09_005308	8 × 10^−60^	52
	Tup1	K4I79_004140	4 × 10^−24^	50
Immune evasion	Hgt1	K6H10_003783	2 × 10^−169^	92
	Msb2	K6H11_002598	2 × 10^−158^	74
	Pra1	CTRG_03753	1 × 10^−136^	74
Phospholipase and proteinase	Lip5	K6H10_004453	2 × 10^−129^	65
	Lip6, Lip8	CTRG_04188	0	75
	Lip7	K4I79_002243	5 × 10^−147^	68
	Sap1-3	SAPT1	2 × 10^−149^	70
	Sap5	SAPT4	3 × 10^−133^	68
	Plb1	K4I79_005789	3 × 10^−77^	70
	Plb2	K6H10_000299	0	83
	Plb3, Plb5	CTRG_05889	0	89
Thermotolerance	Hsp60	K6H10_005454	0	97
	Hsp104	K6H09_002392	0	96
	Ssa1	No found	-	-

* Protein nomenclature corresponds to accession codes of the National Center for Biotechnology Information database (https://www.ncbi.nlm.nih.gov/). The putative protein sequence encoded by the *C. albicans* gene was subjected to a standard protein BLAST (version + 2.17.0) analysis at https://www.ncbi.nlm.nih.gov/. ** When comparing the encoded protein of *C. tropicalis* with the putative ortholog in *C. albicans*. The best hit is reported in the *C. tropicalis* protein column, and this was scored with the lowest E value. The similarity column refers to the comparison of amino acid sequences from the *C. albicans*-encoded protein and the best hit.

## Data Availability

No new data were created or analyzed in this study. Data sharing is not applicable to this article.
